# Baboon Feeding Ecology Informs the Dietary Niche of *Paranthropus boisei*

**DOI:** 10.1371/journal.pone.0084942

**Published:** 2014-01-08

**Authors:** Gabriele A. Macho

**Affiliations:** Research Laboratory for Archaeology (RLAHA), Oxford, England; Universidad Autonoma de Barcelona and University of York, Spain

## Abstract

Hominins are generally considered eclectic omnivores like baboons, but recent isotope studies call into question the generalist status of some hominins. *Paranthropus boisei* and *Australopithecus bahrelghazali* derived 75%–80% of their tissues’ δ^13^C from C_4_ sources, i.e. mainly low-quality foods like grasses and sedges. Here I consider the energetics of *P. boisei* and the nutritional value of C_4_ foods, taking into account scaling issues between the volume of food consumed and body mass, and *P. boisei*’s food preference as inferred from dento-cranial morphology. Underlying the models are empirical data for *Papio cynocephalus* dietary ecology. *Paranthropus boisei* only needed to spend some 37%–42% of its daily feeding time (conservative estimate) on C_4_ sources to meet 80% of its daily requirements of calories, and all its requirements for protein. The energetic requirements of 2–4 times the basal metabolic rate (BMR) common to mammals could therefore have been met within a 6-hour feeding/foraging day. The findings highlight the high nutritional yield of many C_4_ foods eaten by baboons (and presumably hominins), explain the evolutionary success of *P. boisei,* and indicate that *P. boisei* was probably a generalist like other hominins. The diet proposed is consistent with the species’ derived morphology and unique microwear textures. Finally, the results highlight the importance of baboon/hominin hand in food acquisition and preparation.

## Introduction

*Papio* and *Theropithecus* are considered good analogues for an assessment of the adaptive suite of hominin dento-cranial and manual morphology relating to the lineages’ dietary radiation and the ecological drivers underlying it [1]–[3]. Hominins and papionins have evolved under the same ecological conditions in East Africa, have been sympatric and synchron throughout their evolutionary history and exhibit broadly comparable pulses of speciations and extinctions [4], [5]. Together with suids, hominins and baboons presumably shared the same dietary niche [6]. Except for *Theropithecus oswaldi* and its extant relative, *Theropithecus gelada*, papionins are selective omnivores [1], [2], [7], [8], that is “… these animals are neither lawn mowers, chewing up everything in their pathway, nor statisticians, taking random samples.” [9,p. 312]. The composition of baboon diet differs between groups and individuals as a result of local habitats, seasonal fluctuations in resources and individual preferences [7], [10]. This flexibility and selectivity enables baboons to extract the maximum amount of energy and nutrients from the foods available, even when the environments appear resource poor while, concomitantly, limiting the intake of tanins and excessive amounts of fibers; unlike grazers, baboons lack the gut physiology to digest large amounts of fibers [11], [12], [13]. By employing a selective feeding strategy, short-term, e.g. seasonal, fluctuations in resources can therefore be buffered [7]. This is important for a large-brained, slow-growing primate [14], [15], as brains are expensive to grow and to maintain and require a constant supply of energy-rich foods [16]. With this in mind, large-brained hominins are expected to have been selective feeders too. Yet, isotope analyses imply that at least 2 early hominins, *Paranthropus boisei* from East Africa and *Australopithecus bahrelghazali* from Chad, spent some 75–80% (up to 91%) of their time feeding on grasses and sedges [17]–[20]; these foods are generally considered low-quality [17], [19], [21]. Neither hominin dento-cranial morphology nor broader biological considerations are consistent with such a grazing low quality diet. Here I explore whether the energetic requirements of *P. boisei* could have been met by a C_4_ diet, and bearing in mind the limitations of *P. boisei* dento-cranial morphology. The volume of C_4_ foods consumed by yearling baboons [9] is first scaled up to account for the larger body masses of hominins, and the respective nutritional yields are calculated. Adjustments are made to account for the greater manipulatory capabilities of adult baboons (and hominins) for the extraction and processing of corms. By varying the time allocated to eating various C_4_ sources, I then enquire how many minutes per day a 34–49 kg *Paranthropus boisei* [22] would have had to feed on C_4_ sources to meet approximately 80% of its daily energy requirements.

## Materials and Methods

In a landmark study, Altmann [9] meticulously recorded the feeding ecology of yearling, i.e. weanling, baboons (*Papio cynocephalus*) from the Amboseli National Park, Kenya, including the basic nutritional values of these foods (Tables S1, S2 in File S1); similarly detailed information is not available for adult baboons. However, evidence suggests that the foods consumed by adults differ little from those of yearling baboons [7]. Data for immature baboons were thus considered appropriate to serve as a template for the models against which the feeding ecology of *P. boisei* was assessed. Only plants that could be identified as following the C_4_ photosynthetic pathway were selected [23]–[25]; C_3_ foods were not considered. Although not identified as one of the core foods in Altmann’s study, grasshoppers are eaten by baboons also and were included in the models, whereby the time feeding on them was set equivalent to that for dung beetles. As invertebrates may generally have played a significant role in the diet of hominins [26] and vertebrates were not included in the model at all, the outcomes for animal sources are likely an underestimation though.

**Figure 1:**
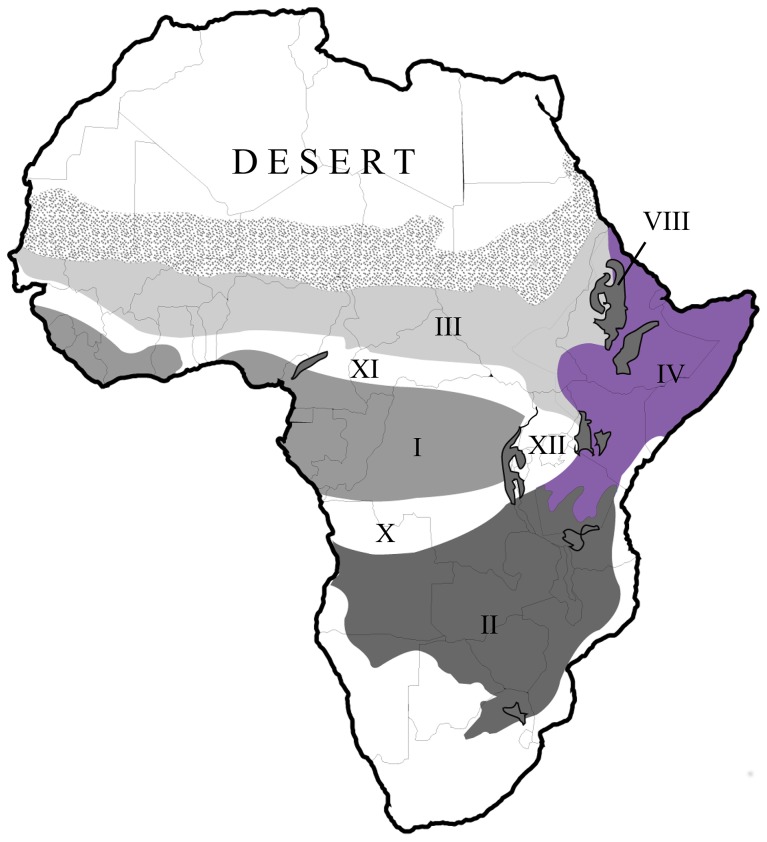
Map of Africa showing the phytogeographical zones, adapted from [27]. The phytogeographical zone IV (Somalia-Masai steppe and shrubland) was occupied by *P. boisei* and is now occupied by the *Papio cynocephalus* population used in this study. I Guineo-Congolian humid forest, II Zambezian miombo woodland, III Sudanian woodland, IV Somalia-Masai steppe and shrubland, X–XII transition mosaic of forest/savanna/woodland, VIII Afromontane domain. The location of the *A. bahrelghazali* sites in Chad falls outside these recognised zones (stippled). Hence, no attempt was made to more accurately assess the possible dietary ecology of this species.

The choice of immature baboons from the Amboseli as a template for adult hominin feeding ecology is justified on 2 grounds: First, nutrient requirements of immature primates are proportionally higher than they are for adults and may therefore be more comparable to the requirements of a larger-brained hominin than the diet of adult baboons would be. Second, the Amboseli National Park lies within the same phytogeographical zone as the *P. boisei* sites (Figure 1). This vegetation zone is dominated by Poaceae grasses and Cyperaceae sedges. The characteristics of the habitat have apparently changed relatively little since 2.7 Ma [27]. Hence, an understanding of whether, and how, yearling baboons extract high-quality foods from this seemingly impoverished habitat directly informs hominin evolution.

The volume of food consumed increases with body mass. Here I use the scaling factor determined by Ross et al. [28] whereby the volume of food consumed scales to body mass as V_d_ = 3.676 M_b_^0,919^. The values are related to the 2.27 kg yearling baboons (average body mass) for whom the amounts of food eaten and the feeding times are known [9, Tables S1, S2 in File S1]. Yearling baboons, like adult baboons, spend considerable time feeding on corms (53 minutes) but, due to their lack of skills and physical strengths, find it difficult to extract corms from the ground and to subsequently process, i.e. clean and peel, them prior to ingestion [9]. In contrast, adult *P. ursinus* from the Drakensberg, South Africa, who extenstively feed on corms on a seasonal basis, have been observed to efficiently extract corms by pulling bundles of grasses from the ground [11]. To account for the inefficient harvesting capabilities of immature baboons, a scaling factor for corm manipulation (m) was therefore introduced. Adult baboons are assumed to double the rate of processing time per minute (*B_j_*) compared to yearling baboons (m = 2) (Table S1 in File S1). The effects of higher scaling factors, i.e. 2.5 and 3 times, were also explored.

Time spent feeding on C_4_ sources was increased incrementally by 10 minutes from the yearling baboon baseline, i.e. some 88 minutes per day (202 kJ). The relative proportion of foods within each subset analysed (e.g., different kinds of corms, or fruits etc.) was retained in each model. The nutritional yield of the various models was outputted and was assessed against the animal’s energetic requirements, its overall time budget for feeding and foraging, and the constraints imposed by its dento-cranial morphology.

**Figure 2:**
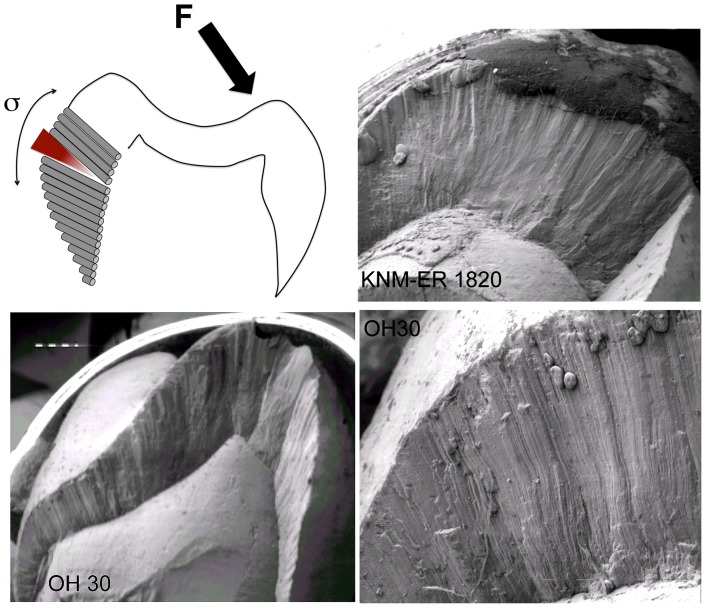
Illustration of the tensile stresses (σ) and resulting breakages in *P. boisei* teeth. Tensile stresses (σ) would occur when lateral loads are applied to a straight-walled tooth and the force vector is directed outside the dental tissue. Without decussating enamel, i.e. bundels of enamel prisms crossing over, transverse cracks initiated on the unloaded side will propagate through the tissue and will lead to catastrophic failure of the tooth. Cracks tend to travel along the protein-rich prism sheaths and are stopped by differently-oriented prisms. Such oblique/transverse breakages are frequently found in *P. boisei* teeth and are illustrated here in a sample of SEM pictures. Although these breaks may have occurred post mortem, they illustrate the plane of least resistance and thus allow an assessment of the loading conditions to which the tooth should not have been subjected *in vivo*. Images are not to scale and are for illustration only.

*Paranthropus boisei* dental micro-morphology is ill equipped to dissipate the laterally-directed loads that would occur while shearing tough foods (Figure 2): it largely lacks enamel prism decussation, which provides the structural strengthening to the tissue that acts as a crack-stopping mechanism [29], [30]. Decussation is brought about by the undulating/sinusoidal 3D paths of ameloblasts from the dentino-enamel junction to the outer enamel surface [31]; the amplitude and frequency of this wavy path generally decreases as the prisms approach the outer enamel surface [32]–[34]. Consecutive layers of prisms are slightly off-set with regard to the onset of this curve, largely due to a delay in onset of ameloblast activity (i.e., extension rate). This results in layers of prisms (and the crystal orientations within) being somewhat angled relative to each other, which makes it difficult for cracks to propagate easily through the tissue [35]. Although differences in individual prism paths between species appear subtle [33], the combined effects of these prism undulations along and between layers of prisms are remarkable, as can be appreciated from naturally broken surfaces (Figure 2, Figure S1 in File S1). They are species-specific. The biomechanical consequences are significant too [36]–[39]. Loading of parallel-oriented prisms, as in the case of *P. boisei*, would result in high tensile stresses between prisms when loaded at a high angle relative to the long axes of prisms [38], which would render the tooth vulnerable to transverse fractures [30] (Figure 2). To account for this limitation of *P. boisei* teeth, models were created where the feeding time was increased for those C_4_ foods only that are well suited to be broken down by the masticatory apparatus of *P. boisei* (i.e. those that require mainly vertical forces): hard, brittle or soft. These foods include corms [40], fruits, flowers and invertebrates (it is acknowledged that some C_4_ fruits may not be soft). No adjustments were made for the large tooth crown areas of *P. boisei* [41] and, presumably, greater processing capabilities. The models created deliberately aim to give a conservative estimate of the dietary ecology of *P. boisei*.

**Figure 3:**
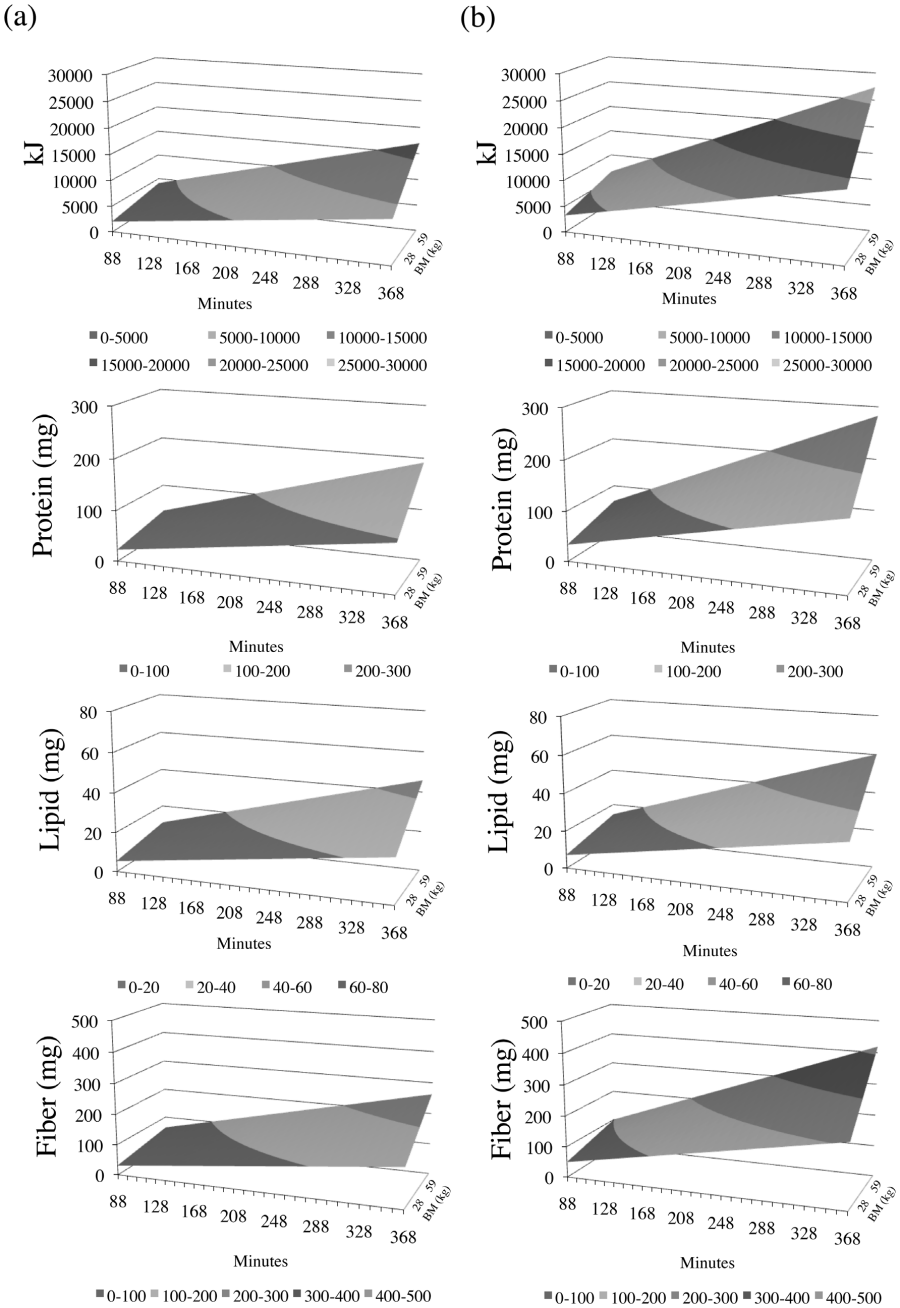
Changes in nutritional yield (*y*-axis) as a result of increasing food volume with body mass 28–59 kg (*z*-axis) at 10-minute increments of feeding on C_4_ sources (*x*-axis) for yearling baboons (a) and yearling baboon with increased manipulatory capabilities m = 2, i.e. a doubling the efficiency (*B_j_*) with which they process corms (b). Incremental steps are highlighted by shaded bands.

**Figure 4:**
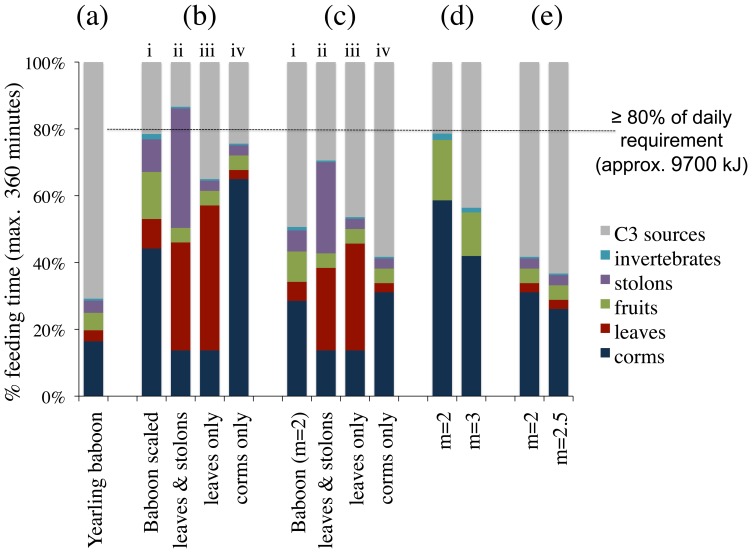
Summary diagram of the composition of diet eaten by a 34–49 kg hominin. In (a) the empirical data for yearling *Papio cynocephalus* are shown. In (b) the basic model shown in (a) is scaled up to account for larger body masses and feeding on all C_4_ sources is increased until the target of approximately 9700 kJ is reached (i). Then, once the model has been scaled to larger body masses, only the time feeding for stolons, leaves, meristem and seeds is increased (ii.), or on leaves (iii) or corms (iv); feeding time on fruits and invertebrates was kept constant to the level of yearling baboons (ii–iv). In (c) the models outlined in (b) are repeated with improved manipulations skills for the processing of corms (m = 2). In (d) only C_4_ food sources that are well-suited to be broken down by *P. boisei* dento-cranial morphology, i.e. hard, brittle or soft, are selected. The effects of manipulatory capabilities (m) were tested. The models shown in (e) are considered most appropriate for inferences about the feeding ecology of *P. boisei.* These are achieved when all C_4_ sources are selected, but only feeding time on corms is increased beyond the time observed in yearling baboons. The total time available for feeding, including foraging, is assumed to be 50% of the day in all models, i.e. 360 minutes.

The energy requirements of *P. boisei* were calculated using Coelho’s energetic model for daily expenditure *DEE* (kcal 24 h^−1^) [42], [43].
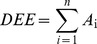
where *A_i_* is the energetic cost (kcal) of an individual activity ‘*i*’ and



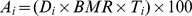
where *T_i_* is the percentage of the day spent performing an activity *i* and *D_i_* is the energy constant for each activity, in this case: D_sleep_ = 1, D_rest_ = 1.25, Df_eed_ = 1.38, D_social_ = 2.35 [44]. As limb lengths for *P. boisei* are not known, the energetic cost of locomotion *A*_loc_ (kcal) was calculated using the generalised mammalian equation [45] together with the average time budgets of adult Amboseli baboons during the dry and wet seasons [46].



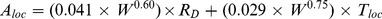
where *W* is body mass, *R*_D_ is the day range (km) and *T_loc_* is the time spent moving. Body mass estimates from fossil remains are contentious [47], therefore the energetic requirements across the entire body mass range of *P. boisei,* i.e. 34–49 kg [22], was calculated. The *DEE* calculated here is some 5% above 2 × BMR, where BMR = 354 *W* ^0.75^ per day [9]. Hence, the energetics calculated can be considered reasonable, as *DEE* is commonly regarded to fall between 2–4 times the BMR [48]. Models that maximise the energy, protein and lipid return while, at the same time, minimise the fiber content are regarded most desirable [49]. The effects of feeding time, body mass and increased manipulatory skills on nutritional yield are shown in Figure 3, while the summary results for the different hominin-specific models are presented in Figure 4.

The Trustees of the National Museums of Tanzania and Kenya, Meave Leakey, Emma Mbua and Cassian Magori kindly granted access to fossil specimens in their care, and Fernando Ramirez Rozzi loaned me casts of Ethiopian *Paranthropus* specimens for inspection.

## Results

Yearling baboons depend heavily on their mother’s milk [9], but feed some 88 minutes per day on 21 different C_4_ foods, which vary in material properties and nutritional value (Table S1 in File S1). Despite their underdeveloped masticatory apparatus, lack of manipulatory skills and physical strengths, they dedicate 53/88 minutes to feeding on corms (Table S1 in File S1). Scaling the volume of food consumed to larger body masses (28–59 kg) and incrementally increasing the time allocated to C_4_ foods (Figure 3) results in a nutritional yield that would be sufficient to support a 34–49 kg hominin with some 9700 kJ in 283 minutes (Figure 4b [i.]). Fruits and invertebrates are however limited and/or available only seasonally. Hence, feeding time on these sources was constrained to the level of yearling baboons before the effects of other foods on overall nutritional yield and time budgets were assessed. When the feeding time on only leaves and stolons is increased from the baseline, the target of 9700 kJ cannot be met within the total feeding/foraging time allocated: the animal would have to feed some 312 minutes on C_4_ sources. Lengthening the feeding time on leaves improves the result over the basic model (234 minutes), as would preferential feeding on corms (272 minutes). While the former model is problematic on mechanical grounds [50], the latter is unrealistic because yearling baboons, unlike adults, have inadequate manipulation skills to extract and process corms [9]. To account for the greater manipulatory skills of adult baboons or hominins, the yearling baboon processing time for corms/_minute_ (*B_j_*) was doubled (m = 2), and the analyses were repeated (Figure 4c). As above, (i.) presents the general scaled-up model, while models (ii.)-(iv.) are constrained with regard to fruit and invertebrate intake. Introducing improved manipulation skills results in the target of 9700 kJ being achieved in 178 minutes, i.e. 50% of maximum feeding/foraging time per day (Figure 4c [i.]). Figure 3 illustrates the steep rise in energy (kJ) output with improved manipulation, which is followed by lipids and protein; the increase in fiber content is less pronounced. This is advantageous as fiber constitutes a constraint on baboon size [51] and, by inference, hominins [49]. A preferential increase in feeding on leaves/stolons/meristems and seeds, or leaves only, increases total feeding time, but a preferential increase in corm time decreases the total time to 140 minutes, i.e. 42% (Figure 4c).

To test whether *P. boisei* may have been a dietary specialist, models were then created that included only hard, brittle and soft foods, i.e. all potentially tough foods were excluded. For feeding time on C_4_ foods to fall under 57% (203 minutes) of the total time budget, manipulation skills would need to be increased to 3 times that of a yearling baboon (Figure 4d), while the intake of fruit would need to be unacceptably high. A preferential increase in corm time, which leaves the time for feeding on fruits, flowers and invertebrates at the level of yearling baboons, substantially changes the nutritional yield of the diet, whereby the amount of lipids and proteins decreases and fiber content increases (Figures S2–S5 in File S1). These specialised models are therefore deemed unsuccessful.

A generalised baboon model (Figure 4e) that includes all of the C_4_ foods preferred/eaten by yearling baboons, but only increases the feeding time on corms beyond the level consumed by yearling baboons yields the most favourable results, both in terms of time budget and nutritional yield (Figure 4e, Table S3 in File S1). Such a diet would be consistent with *P. boisei* dental morphology: because of their immaturity, the mechanical properties of foods consumed by yearling baboons are not considered particularly demanding, with the exception of corms [9], and hence would have been suitable for *P. boisei* also. Depending on the manual skills for corm extraction inputted, i.e. 2 or 2.5 times that of a yearling baboon, *P. boisei* would have needed to feed some 150 minutes (corms: 112) or 133 minutes (corms: 94) on C_4_ sources in order to obtain 9700 kJ. This translates to about 42% and 37% of total daily feeding/foraging time. This value falls sharply below the 75%–80% implied by isotope studies. Importantly, the time-budget calculated would enable an animal to comfortably meet the higher energetic demands of 2–4 times the BMR that regularly occur because of additional costs relating to thermoregulation, predator defence, reproduction etc. [48].

## Discussion

*Paranthropus boisei*, with its highly derived dento-cranial morphology, remains one of the most enigmatic hominins. Suggestions range from masticating hard small objects [40], [52], repetitive chewing [53], habitual consumption of soft material [54] and feeding on abrasive grasses [17], [55]. Not all proposals are compatible with the species’ morphology though (Figure 2). More importantly, the implied dietary specialisation (i.e., stenotopy) is not supported by other evidence [56] or by general considerations about hominin palaeobiology and life history [16]. As *P. boisei* was a highly successful taxon, spanning over 1 myrs and living through fluctuations in the physical environment [57], it is unlikely to have lived on the brink. The results of the present models imply that *P. boisei* could have obtained sufficient nutrient-rich foods within the constraints of its daily time budget for foraging and feeding.

Ascertaining the diet of an extinct species is imprecise at best, and the present study does not pretend otherwise. Rather, the outcomes of the models are of heuristic value as they aim to determine whether a medium-sized large-brained hominin could have subsisted on a predominantly C_4_ diet. Such a diet must combine a number of prerequisites: (i.) being readily available within the environment, (ii.) being predominantly made up of the material properties to which the masticatory apparatus of *P. boisei* (or other hominins) is adapted, (iii.) being of sufficient nutritional value to support this hominin but without an excessive fiber load and (iv.), be harvestable within the time budget available. By selecting food sources available within the specific environment and by modifying the empirically derived data of extant baboons *Papio cynocephalus* [9] this can be achieved. Scaling issues and the nutritional diversity of C_4_ foods must however be given due regard when reconstructing the dietary ecology of hominins.

The volume of food consumed as well as feeding time increases with body mass [28]. Foods vary in energy and nutrients, and the amount ingested per minute varies between foods [9]. An increase in volume, whether due to body mass, dietary preference or both, will therefore automatically change the total dietary composition and nutritional yield of that diet, simply because the component parts of the diet do not change isometrically with volume. As a case in point, an increase in feeding time on corms increases the nutritional yield more dramatically than an increase in feeding on grasses by the same length of time (provided the manual dexterity of adult baboons/hominins is taken into account). For this reason it is possible for a medium-sized primate to obtain 80% of its daily requirements whilst spending relatively little time feeding on C_4_ sources (Figure 4). The relatively low values of 42%–37% suggested for *P. boisei* (Figure 4e) are probably an overestimation still. First, no attempt was made to account for the masticatory capabilities of *P. boisei* as reflected by their large tooth crown areas [41]. Second, the manipulatory skills used are only moderate improvements over the capabilities of small-sized (2.27 kg) yearling baboons. Third, data are forthcoming that suggest that (at least some) corms increase their oil content as they mature, while protein and sugar levels decrease [58]; this would increase the overall energy return. Taken together, a time budget closer to 30% may be more realistic for *P. boisei*. Either way, the relatively high corm content would provide this hominin with high amounts of minerals and vitamins [59], including important fatty acids [60], [61]. Importantly, such a diet is compatible with the derived dento-cranial morphology of *P. boisei*, and its dental wear patterns.

Both macro- and microwear patterns of *P. boisei* teeth support propositions that *P. boisei* included a large proportion of corms in its diet. Corms are rich in starches (up to 50%), which are highly abrasive in unheated state and vary in size [*C. esculentus*: 3–12 µm [62]; *C. rotundus:* 30–110 µm [63]]. Starches are not broken down mechanically though, but chemically through the interaction with amylase contained within saliva [64]; lengthy oral processing would facilitate this process. Unsurprisingly, the rates of wear of Amboseli baboons correlate with corm consumption [65]. The thick enamel of *P. boisei* teeth is almost certainly an adaptation to wear resistance [66], while the flatly worn tooth surfaces bear direct witness to the milling process [67], [68], which results in “polished” wear surfaces, i.e. indistinct microwear textures [54]. It is not necessary to invoke agents other than starches to account for *P. boisei’*s unique macro- and microwear patterns. Repetitive chewing (rather than high bite forces) would have been advantageous, and has been inferred on the basis of the species’ musculature [53] and its unique temporo-mandibular joint morphology that emphasises lateral pterygoid muscle pull [69], i.e. the transverse movement of the mandible. Although all baboons eat and prefer corms, sometimes in considerable quantities [11]–[13], [70]–[73], they vary the intake on an inter-annual basis. This seasonal variation in consumption of C_4_ corms is expected to dampen the isotopic composition of baboons’ tissues, although some populations were reported to have exceptionally high δ^13^C values [74]. For *P. boisei*, in contrast, corms probably constituted the main staple food which, given their physico-chemical properties, conceivably selected for the species’ unique dento-cranial morphology (and bearing in mind the larger quantities consumed due to body mass scaling alone). As is the case for baboons [7], regional, individual and seasonal variations in diet are however expected, as implied by isotope results also [17]. What is noteworthy is that exclusive reliance on only one food source seems unlikely though (as it would be for other hominins).

Foods vary in fiber content, tanins etc. and selective omnivores, like hominins [75], must find an optimal balance between various foods [7]–[9]. Determining the optimal composition of a primate diet is not trivial [76]. Underlying this work is the assumption that yearling baboons “know” what to eat, i.e. intuitively select food according to their needs, and that the nutritional requirements of a hominin may not differ much. This assumption seems justified, as a radical change in the composition of the diet, i.e. leaving out some foods altogether (Figure 4d, Figure S2–S5 in File S1), resulted in a noticable dietary imbalance. Although not necessarily detrimental, provided the time budget allows for the supplementation of important nutrients from C_3_ sources (with the required material properties), such models should be viewed with caution. The more inclusive models presented in Figure 4e fulfill both the (assumed) nutritional and the time-budget requirements, and are thus considered more appropriate proxies for the dietary ecology of *P. boisei*.

Stable isotope analyses are a useful tool for the reconstruction of the dietary niches of hominins [18]. But not all C_4_ foods are low quality. Hominins, like baboons, are likely to have been selective in their food choice. Which C_4_ foods were habitually consumed can only be determined on the basis of morphology, including body mass and brain size, and in conjunction with an animal’s energetic requirements. *Theropithecus oswaldi*, *P. boisei* and *A. bahrelghazali* are comparable in isotopic composition [17]–[20], yet their diets most certainly differed. Only *Theropithecus* exhibits the morphological features commonly associated with graminivory that include *inter alia* hyposodont thin-enamelled teeth with shearing crests [4], [5] and high levels of prism decussation [77], and absolutely and relatively smaller brains compared with *Papio* [78]. Hominins differ, even among themselves. Unlike *P. boisei*, *A. bahrelghazali* teeth are buttressed and relatively thin enamelled [79]. Excessive consumption of corms can therefore be ruled out and a diet of predominantly tough foods is implicated. If confirmed, this may indicate that, although morphologically more generalised than *P. boisei*, *A. bahrelghazali* could have been more specialised behaviourally. Regardless, on the basis of the present analyses it is suggested that *P. boisei*, like extant *Papio*, was a dietary generalist, albeit with a preference for corms. It probably was an ecological generalist too. Despite feeding predominantly on savanna C_4_ foods, *P. boisei* appears to have occupied fairly wooded well-watered environments [80]–[82], where corms are known to thrive. This eurybiomic strategy seems to underlie the evolutionary success of *P. boisei*. With the disappearance of deep-water lakes and the onset of an arid cycle at about 1.45 Ma [83] the availability of corms would have declined, while competition with *Papio* and the more encephalized *Homo* for alternative resources would have increased. These factors, either in isolation or in combination, are probably responsible for the demise of *P. boisei.*

## Supporting Information

File S1 **Supporting figures and tables.** Table S1 Primary data used to create the models. Table S2 Summary of the nutritional yield of each food category used. Figure S1 Scanning electron microscope images of naturally broken teeth of hominins illustrating differences in prism decussation. Figure S2–Figure S5 Differences in kJ, protein, lipid and fiber yielded in the specialised models when different scalars are used for manipulatory skills.
(DOCX)
