# Supplementary material for: Baboon Feeding Ecology Informs the Dietary Niche of Paranthropus boisei
Source: PLoS One. 2014 Jan 8;9(1):e84942. doi: 10.1371/journal.pone.0084942 (PMC3885648; doi:10.1371/journal.pone.0084942)
Supplement: File S1 — Supporting figures and tables. Table S1 Primary data used to create the models. Table S2 Summary of the nutritional yield of each food category used. Figure S1 Scanning electron microscope images of naturally broken teeth of hominins illustrating differences in prism decussation. Figure S2–Figure S5 Differences in kJ, protein, lipid and fiber yielded in the specialised models when different scalars are used for manipulatory skills. (DOCX) [file pone.0084942.s001.docx]

**Supporting Information**

**Table S1** The primary data underlying the creation of the models are derived from Altmann (1998) for yearling baboons. The term “manipulation (m)” used in the main text refers to the units processed per minute (*B*_j_): this value was manipulated for corm processing (see text). This step was taken as adult baboons (and hominins) have greater strength to extract these below-ground sources, as well as greater manipulatory skills to process them extra-orally. Minutes of feeding per day (*A*_j_) were incrementally increased by 10 minutes, distributed across all resources (or a subset thereof), thus leaving the relative proportion of each food item unchanged. The categories used are corms (C), leaves (L), stolons (S), fruits (F) and invertebrates (I). Note that meat was not included in the model although it plays a role in baboon, and presumably hominin, diet.


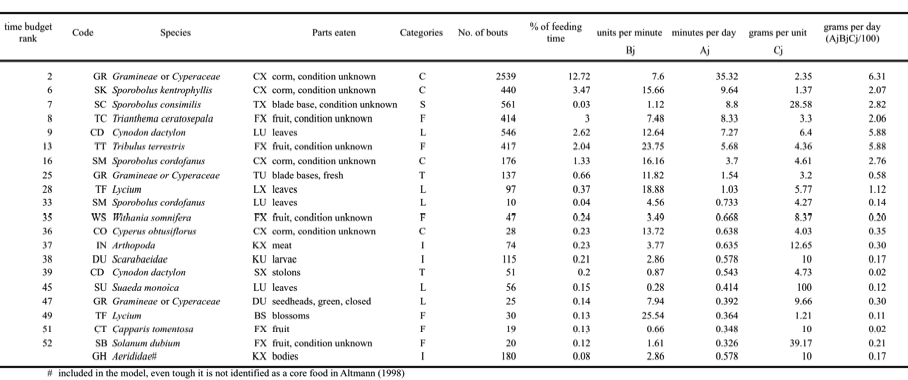


**
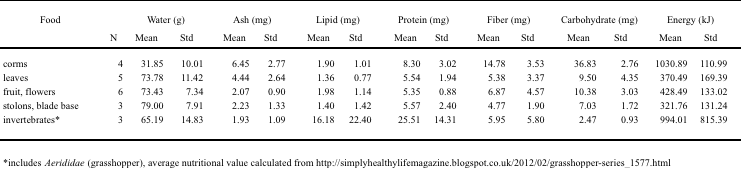
Table S2** Summary of the nutritional yield for each food category.

**Figure S1** Scanning electron microscope images (SEM) of naturally broken enamel structures of hominin teeth. (a) *Australopithecus afarensis* (LH6, I), (b) *Kenyanthropus platyops* (WT38356, RM1/2), (c) *Australopithecus africanus* (Stw208, RM1) and (d) early *Homo* from Swartkrans, South Africa (SKX269, RC). No image is provided for *P. robustus,* because it is not possible to put the entire width of the broken surface into focus within a single image, owing to this species exhibiting a marked sinusoidal curve in the transverse plane also. Arrows are placed at the dentino-enamel junction and point towards the cusp tip. For *A. afarensis* (a) and early *Homo* (d) images are from more cervical regions, which explains the thinner enamel; all other images are from mid-crown levels. As enamel thickness increases cuspally, so does the level of decussation and it becomes increasingly difficult to appreciate the undulating paths of prisms. However, the basic pattern of undulation (for a given enamel thickness), i.e. frequency and amplitude of prism undulation and the rate at which prisms are offset relative to each other apico-cervically, appear unique to each primate. The different patterns thus resulting can be appreciated with the naked eye. In hominins, levels of decussation vary substantially from high (d) to moderate (b) and low, as seen in *Paranthropus boisei* (see Figure 2, main text). The scale bars generated by the SEM (10 microns) are in white.


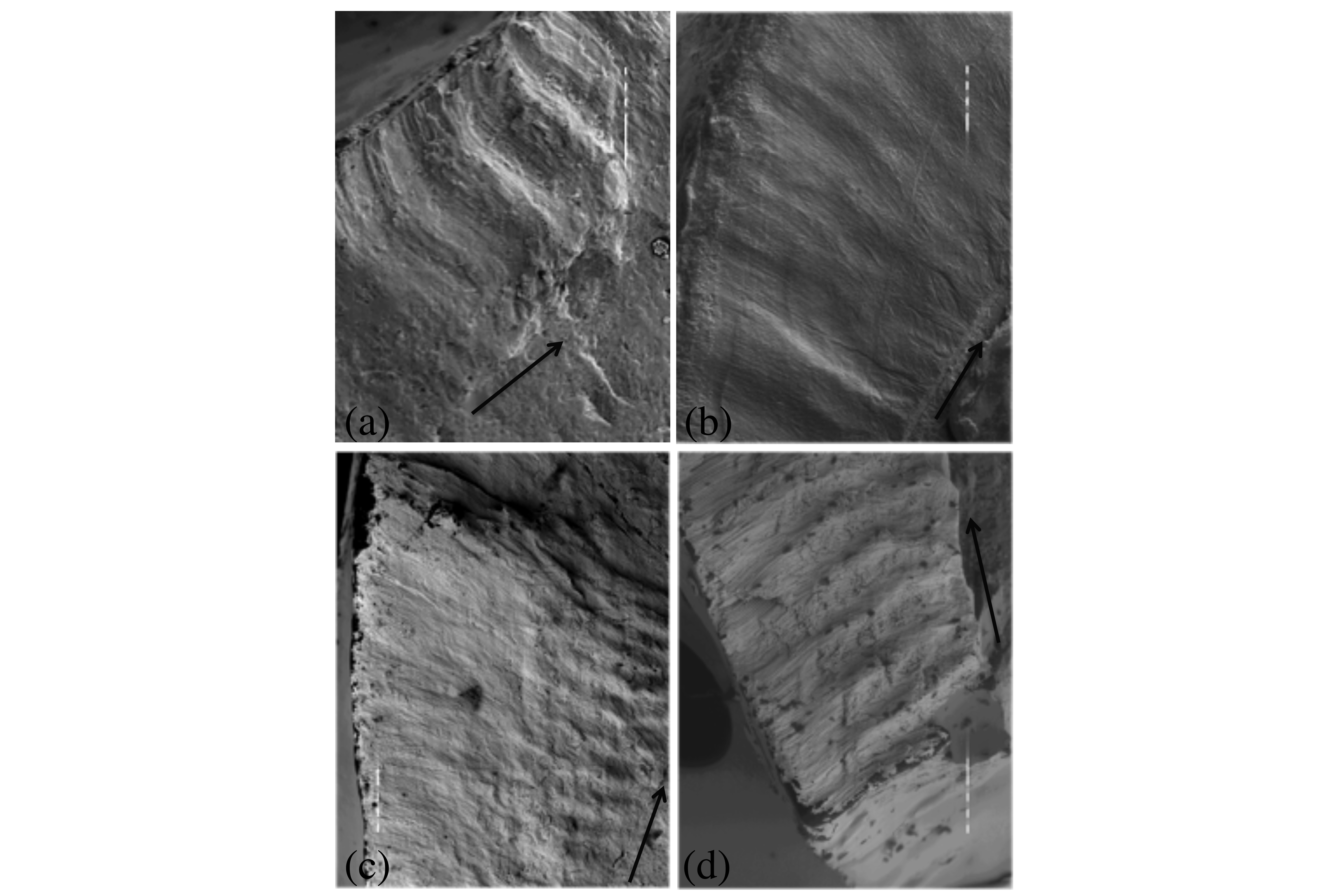


**Figure S2** The effects of body mass 28-59kg (*z*-axis) and increased feeding time on all non-tough foods (a) and corms only, i.e. leaving the other non-tough foods at the level of yearling baboons, (b) on energy output (kJ). The effects of different manipulatory skills (m) are shown.





**Figure S3** The effects of body mass 28-59kg (*z*-axis) and increased feeding time on all non-tough foods (a) and corms only (b) on protein output, using different manipulatory skills (m).

**

**

**Figure S4** The effects of body mass 28-59kg (*z*-axis) and increased feeding time on all non-tough foods (a) and corms only (b) on lipid output, using different manipulatory skills (m).





**Figure S5** The effects of body mass 28-59kg (*z*-axis) and increased feeding time on all non-tough foods (a) and corms only (b) on fiber output, using different manipulatory skills (m).





**Table S3** Nutritional content of the *P. boisei* diet proposed in Figure 4 of the main text.

**
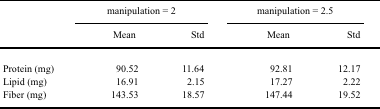
**

**References**

Altmann SA (1998) *Foraging for Survival.* Chicago: University of Chicago Press.
